# Potential Development of N-Doped Carbon Dots and Metal-Oxide Carbon Dot Composites for Chemical and Biosensing

**DOI:** 10.3390/nano12193434

**Published:** 2022-09-30

**Authors:** Yogita Sahu, Ayesha Hashmi, Rajmani Patel, Ajaya K. Singh, Md. Abu Bin Hasan Susan, Sónia A. C. Carabineiro

**Affiliations:** 1 Department of Chemistry, Govt. V. Y. T. PG. Autonomous College, Durg 491001, Chhattisgarh, India; 2 Hemchand Yadav University, Durg 491001, Chhattisgarh, India; 3 School of Chemistry & Physics, University of KwaZulu-Natal, Westville Campus, Durban 4000, South Africa; 4 Department of Chemistry, University of Dhaka, Dhaka 1000, Bangladesh; 5 LAQV-REQUIMTE, Department of Chemistry, NOVA School of Science and Technology, Universidade NOVA de Lisboa, 2829-516 Caparica, Portugal

**Keywords:** N-CDs, metal-based CDs composites, properties, chemical sensing and biosensing, properties

## Abstract

Among carbon-based nanomaterials, carbon dots (CDs) have received a surge of interest in recent years due to their attractive features such as tunable photoluminescence, cost effectiveness, nontoxic renewable resources, quick and direct reactions, chemical and superior water solubility, good cell-membrane permeability, and simple operation. CDs and their composites have a large potential for sensing contaminants present in physical systems such as water resources as well as biological systems. Tuning the properties of CDs is a very important subject. This review discusses in detail heteroatom doping (N-doped CDs, N-CDs) and the formation of metal-based CD nanocomposites using a combination of matrices, such as metals and metal oxides. The properties of N-CDs and metal-based CDs nanocomposites, their syntheses, and applications in both chemical sensing and biosensing are reviewed.

## 1. Introduction

The rapid increase in the world population has caused a sharp rise in environmental pollution. The most alarming example is water pollution involving the scarcity of potable water due to the discharge of various types of pollutants such as phenolic compounds, inorganic materials, heavy metals, pesticides, and pathogenic microorganisms [1,2,3], that come from industries such as paper, printing, food processing, pharmaceutical, and cosmetic. This is an increasingly serious problem causing fatal diseases in the world [4,5,6]. The identification of inorganic and organic contaminants in various sectors, such as environmental monitoring, chemical and biological analysis, healthcare, and food safety, is crucial [7,8,9].

Several techniques for monitoring of water quality and detection of contaminants are popular, including conventional instrumental laboratory-based analysis, such as mass spectrometry, electrochemical methods, and gas chromatography. The prospective advantages of the laboratory-based analytical methods have been recognised for a long time, but studies show that they are not very efficient for on-site monitoring applications. With the technical improvements in analytical chemistry, new techniques are available for real-time detection such as, advanced spectroscopy, model-based event detection and water quality sensors [10,11].

In this regard, the introduction of sensor technology is attracting considerable attention. Sensors are classified into chemical, and biosensors based on their nature of sensing including the qualitative and quantitative analysis of a specific type of sample with the help of spectroscopic, electrochemical, and photoluminescence properties of the systems [12]. The chemical sensor involves the detection of metal ions and other molecules in the physical systems such as water resources, while biosensors sense molecules of biological importance [13,14].

In the last decade, a significant amount of attention has been given to “nanotechnology”, particularly in the field of sensors. It is one of the most essential technologies encompassing discoveries and inventions in numerous fields, such as chemistry, biology, medicine, physics, chemistry, and engineering. Richard P. Feynman (1959) first coined the word “nanotechnology” [15]. It is made of two words “nano” and “technology”. The word “nano” originated from the ancient Greek word νᾶνος (nános) meaning dwarf [16]. Thus, nanotechnology is the branch of technology where the manipulation of matter takes place on an atomic and molecular scale within at least 1 to 100 nm. Nanoparticles are the basic tools of nanotechnology. Particles with the size within the nano dimensions are known as nanoparticles (NPs) and the materials consisting of these particles are called nanomaterials [17]. The NPs can be 0D, 1D, 2D, and 3D depending on their shapes. A nanocomposite is a combination of the best properties of two or more different materials, in which one of the components has at least one dimension that is around 10^−9^ m (1 nm) [18,19]. In this regard, carbon materials, including fullerenes, nanofibers, graphene oxide (GO), and other carbonaceous nanomaterials have drawn significant attention due to a number of notable characteristics, such as electrical conductivity, chemical reactivity, and photocatalytic properties [20].

In recent years, carbon-based sensors have been reported to play a crucial role in chemical sensing and biological sensing. Carbon “quantum dots” (CQDs) or carbon dots (CDs) represent the carbonaceous family with incredible properties and advantageous features to overcome the shortcomings of common traditional metal-based quantum dots (QDs) such as metal oxides (TiO_2_, ZnO), and inorganic QDs (ZnO-PbS). Their shortcomings include high-carcinogenic toxicity, reactivity, cost, and low biocompatibility, hindering their extensive use and making them responsible for serious health and environmental problems [21,22]. Generally, CDs are spherical carbon NPs in which carbon atoms are sp^2^ hybridised with diameters of less than 10 nm [23,24]. CDs have a large number of -OH and -COOH and -NH_2_ and other groups on their surfaces, which give them outstanding photophysical and chemical properties, together with excellent biocompatibility, negligible toxicity, short response times, considerable aqueous solubility, cell-membrane permeability, and cost effectiveness with good quantum yield (Φ) [25,26,27]. Hence CDs show great importance in different fields, including bioimaging [28], energy harvesting [29], biosensing [30], drug delivery [31], and photocatalysis [32]. The efficiency of CDs significantly improves with the introduction of a heteroatom (N, S, B, P, F, Br, etc.) or transition metal (Zn, Cu, Mg, Ag, Au, etc.) [33,34] through the process of doping and formation of composites of CDs with difference matrices such as metal, metal oxide, polymer, and carbon-based materials (graphene, GO) compared to individual CDs and without CDs [35,36,37,38].

**Figure 1:**
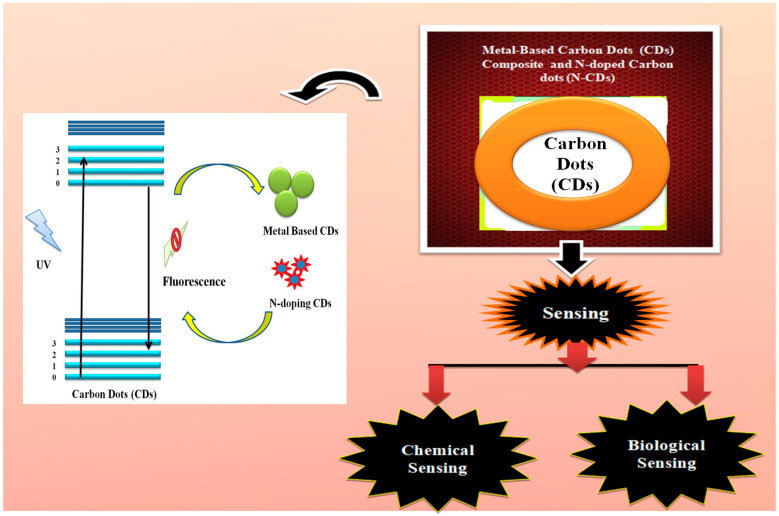
General overview of this work. The potential of CDs as a component of N doping and metal or metal oxide composite materials for chemical and biological sensing is highlighted.

In this review, we describe the sensing abilities of N-doped and metal-based CDs composites in the identification of undesirable components present in physical systems, such as water resources, as well as biological systems (living organisms), which cause pollution and diseases. The interactions of CDs synthesised from different precursors with doped nitrogen and metal or metal oxide to form composites are discussed and the role of the composites for biological and chemical sensing is highlighted. Figure 1 illustrates a general overview of the review.

## 2. Carbon Sources for the Preparation of Carbon Dots

**Figure 2:**
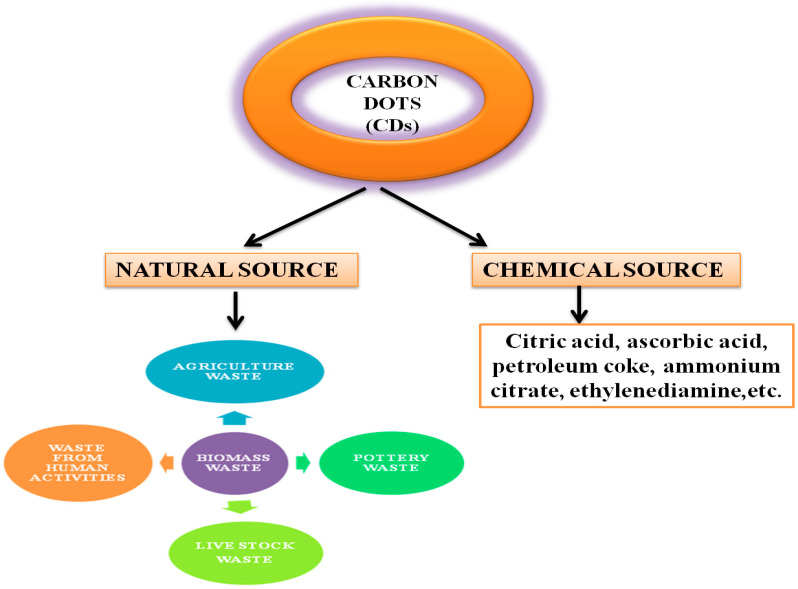
Natural and chemical sources of precursors used in the synthesis of CDs.

CDs can be synthesised from a good number of precursor materials coming from both natural (organic) and chemical (inorganic) sources (Figure 2). The properties of CDs rely on the source materials used for synthesis. For instance, fluorescent CDs exhibit relatively lower Φ when prepared from chemical sources [39], compared to organic carbon sources, such as organic compounds, organic natural products, and biomass waste. Natural sources are more popular for the synthesis of CDs because they are heterogeneous, biodegradable, and bio-organic substances mainly composed of biopolymers such as cellulose (30–60%), hemicelluloses (20–40%), lignin (15–25%), ash, and proteins [40,41]. Renewable, ecofriendly, abundant, and innocuous carbon sources show excellent optical properties and low cytotoxicity. However, most of the natural resources in the form of biomass waste are currently discarded, landfilled, or openly burned, which not only leads to a waste of resources but also causes some environmental problems threatening human life [42,43,44].

## 3. Methods of Preparation of CDs

**Figure 3:**
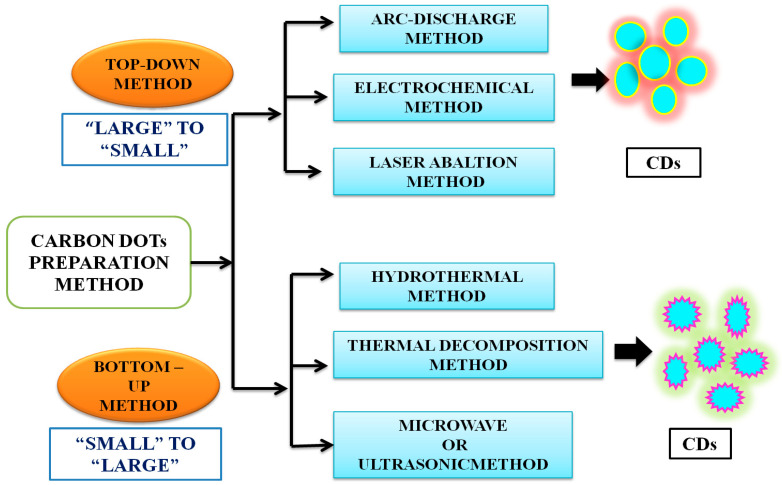
Common top-down and bottom-up approaches to synthesize carbon dots. The details of these methods are available in recent textbooks.

CDs can be prepared by both top-down and bottom-up approaches (Figure 3). The former involves the rupture or cleaving of bulk carbonaceous materials into small materials by physical, chemical, or electrochemical means, including electrochemical synthesis [45], laser ablation [46], arc discharge [47], or chemical oxidation [48]. The top-down method is associated with the advantages of an abundance of raw materials, a large-scale production, and a simple mode of operation. This method, however, also suffers from the drawbacks of low yield, the requirement of special equipment, and use of nonselective chemical cutting method. Thus, the bottom-up method is preferred. It is an easy method and involves carbonization, that is, small molecules are chemically fused in a stepwise way, using microwave [49], ultrasonication [50] and hydrothermal methods [51]. The hydrothermal method has been the most popular method, due to its associated advantages, such as ease and convenience of the procedure, simple operation, cost effectiveness and ecofriendliness, which have rendered it to be considered as a green method [44]. Conventionally, CDs are synthesised by surface functionalization of carbon nanoparticles with organic and polymeric molecules. CDs synthesised using this method often require high-operational temperatures, the use of volatile organic solvents or alkaline and acidic conditions. Sun and coworkers reported the synthesis of CDs by laser ablation of a carbon target, prepared by hot-pressing a mixture of graphite powder and cement, followed by stepwise baking, curing, and annealing, in the presence of water vapor with argon as a carrier gas at 900 °C and 75 k [46]. The treated sample was not photoluminescent, but upon the surface passivation, by attaching simple organic species to the acid-treated carbon particles, typically at 120 °C, for 72 h, bright luminescence emissions could be observed. A similar method was used, and photoluminescent CDs were produced by surface passivation, by Cao et al. [52] and in these cases, the optical properties were widely varying but were less controllable. The experimental conditions also made them environmentally unfriendly [46,52]. Given the natural concerns about environmental sustainability, greener approaches to CD synthesis are urgently needed [53,54].

Both chemical and natural sources are based either on top-down or bottom-up strategies. As noted earlier, the top-down method involves harsh experimental conditions (e.g., strong acid and arc discharge), tedious operation steps, with expensive equipment usually employed, yielding CDs with low Φ, which greatly limits their practical application [55,56,57]. The bottom-up approach is based on the polymerisation reaction of small molecules aiming at the formation of nanoscale CDs. The green synthesis of CDs using the bottom-up approach involves hydrothermal and solvothermal combustion, and template-assisted, microwave, and ultrasonic methods, etc. Among them, hydrothermal methods are the most frequently used due to their advantages, such as low cost, high yields, ease of operation, simple equipment requirements, and their ecofriendly nature [58,59]. The bottom-up synthetic route thus appears promising and is more appealing as it uses “green precursors” (that is, renewable natural resources or their derivatives or precursors made from waste materials of biomass). In contrast, chemical precursors refer to chemical species, such as citric acid, ethylene, etc. [54,60].

CDs can be used as fluorescent chemical sensors for measuring several analytes found in water bodies, such as different types of chemical species such as pesticides and heavy metal ions such as As, Hg, and Cd. CDs find important applications in the quantification of biological molecules and intracellular metal ions (such as Fe^3+^, Hg^2+^, Na^+^, and K^+^), vitamins, enzymes, nucleic acids, proteins, H_2_O_2_, glucose, L-cysteine, and galactose, among others [61,62,63]. The exceptional sensing ability of CDs toward a range of substrates has been exploited and a significant role of CDs was noted in a number of biological processes, such as regulation of metalloenzymes, gene expression, energy generation, and neurotransmission in biological systems [53,54,60].

**Figure 4:**
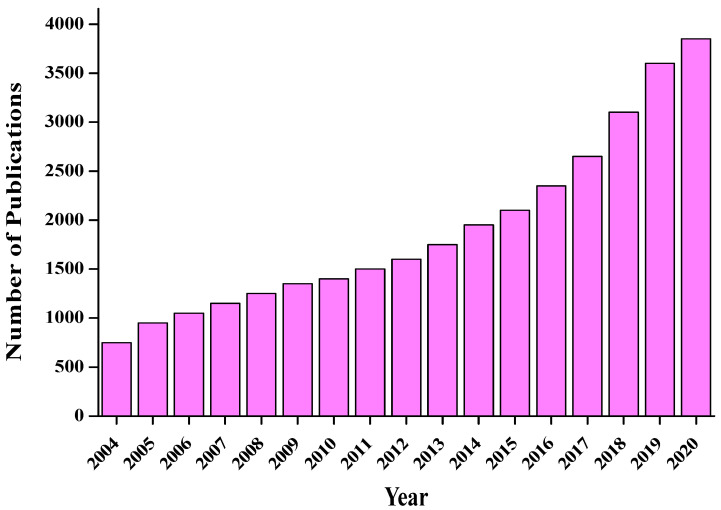
The number of publications related to CDs. A surge of interest in recent years is observed.

CDs are excellent sensors due to their novel characteristics, such as high sensitivity, intrinsic fluorescent properties, low cost, ecofriendliness, quick response, negligible cytotoxicity, and easy preparation method. Thus, they serve as appropriate energy materials or electron acceptors and donors and are a hot topic in modern research. The sensing of CDs generally occurs as a result of a change in their fluorescence behaviour through various means, such as the inner-filter effect, photoinduced electron and charge transfer, and resonance energy transfer [64,65]. Figure 4 displays the progress data for CDs from 2004 to 2020 [66,67]. A nearly exponential rise is observed within the reported time frame.

## 4. Properties of Carbon Dots

### 4.1. Structural Properties

CDs are commonly sphere-like three-dimensional (3D) clusters of 2–10 nm, composed of C, N, O, and H atoms [68]. The proportions of these elements vary, depending on the carbon source used to produce them through different approaches (generally carbohydrates, proteins, and other biomolecules are employed). The inside parts of these clusters are surrounded by mostly sp^3^ hybridised and a small portion of sp^2^ hybridised carbon atoms, along with disordered carbons, which result in an amorphous nature [69,70]. Codoping, surface passivation, and some other treatments improve the water solubility of CDs, allowing more applications [71,72]. Due to low toxicity and biocompatibility, CDs can enter cells without any difficulty via endocytosis, and have extensive relevance in deep-tissue and cell imaging. Raman spectroscopy is widely used for the identification of functional groups and spectral characteristics of the molecular structures of samples [73,74].

### 4.2. Optical Properties

#### 4.2.1. Absorbance

CDs show broad and strong absorption bands in the ultraviolet-visible (UV-vis) region. The absorption spectra vary for CDs synthesised from different sources with various approaches. Water-soluble CDs show a light yellow colour in aqueous solution. Many functional groups present on the surface of CDs cause different types of transitions, such as π-π* transition of C=C and C=N bonds observed at 220~270 nm, while a shoulder peak is observed due to the n-π* transition of C−O and C=O bonds at around 280~350 nm [75,76]. Upon increasing the synthesis temperature, the latter band becomes broader and weaker [77].

#### 4.2.2. Fluorescence Properties

One of the most exceptional features of CDs is their fluorescence properties, which include fluorescence emission, narrow emission, wide excitation, size- or excitation wavelength-dependent fluorescence emission, strong resistance to photobleaching, and upconversion luminescence [78,79]. Zhang et al. [78] reported the influence of organic solvents on the fluorescence emission peak position of CDs. Different organic solvents create different types of defects on the surface of CDs, which initiate different emission sites responsible for intensifying the fluorescence emission spectrum of the materials.

#### 4.2.3. Upconversion Photoluminescence

Upconversion photoluminescence (PL) is one of the most appealing optical features of CDs, affected by surface defects, quantum size, or confinement effects. It is a kind of tunable PL [80]. In tunable PL phenomenon, CDs show excitation-dependent PL along with strong emission, which rapidly decline from blue-wavelength to red-wavelength region. The upconversion phenomenon, on the other hand, involves emission of photon at shorter wavelengths than the excitation wavelength, which is attained by absorbing two or more photons [79,80,81,82]. CDs prepared from the natural sources show special upconversion fluorescence, with an emission wavelength shorter than the excitation wavelength, instead of traditional down-conversion fluorescence [83]. Two types of accepted mechanisms of antistokes photoluminescence and multiphoton active processes are usually used to explain upconversion fluorescence [84]. Sun et al. [85] reported that S and N codoped CDs prepared from hair fibres exhibit upconversion fluorescence behaviour. Wu et al. [86] demonstrated the upconversion phenomenon of CDs prepared from walnut shells by introducing an electronic transition process model. According to this model, the phenomenon occurs due to the gap between the highest occupied molecular orbital (HOMO) and lowest occupied molecular orbital (LUMO). The HOMO–LUMO gap decreases as the particle size of CDs increases. The electrons at the π orbitals (HOMO) of carbine are excited after the absorption of low-energy radiation with wavelengths >600 nm and jump into the excited state with higher energy (LUMO). The electrons in this state return back to the ground σ state and release a huge amount of energy in the form of radiation and, consequently, upconversion luminescence takes place.

#### 4.2.4. Electrochemiluminescence

The electrochemiluminescence (ECL) of CDs plays an excellent role in the clinical diagnosis of intracellular toxic elements, even in ultratrace amounts, due to the high sensitivity and simple mode-of-action of this technique. Some of the numerous novel luminescent materials used so far include iridium complexes [87], fluorescent dyes, and noble-metal nanoclusters [88,89,90]. The conventional techniques for detection, on the other hand, include atomic absorption spectrometry (AAS) [91], electrochemistry [92], fluorescence [93], and inductively coupled plasma mass spectrometry (ICP-MS) [94], which are time consuming. Electrochemical or chemical approaches applied for the synthesis of CDs for ECL applications yield CDs with oxygen-rich functional groups, such as carbonyl (C=O), carboxyl (-COOH), hydroxyl (-OH) and epoxy or ether (R−O−R) [95,96]. These lead to formation of defects and serve as chemically reactive sites, responsible for the degradation of large carbonaceous substances into smaller fragments [97]. While both oxidized and reduced CDs, having different ECL behaviours, are produced using the bottom-up method, negligible or weak ECL behaviour is exhibited by reduced CDs. Strong ECL emission is obtained from oxidized CDs. The oxygen-containing functional groups present on the surface of the CDs produce electrogenerated CDs^•−^ radicals with the help of K_2_S_2_O_8_, where S_2_O8^2−^ produces the strongly oxidizing SO_4_^•−^ radicals by electrochemical reduction. After CDs^•−^ react with SO_4_^•−^ radicals through electron-transfer annihilation, an excited state for ECL emission is generated [98,99].

### 4.3. Cytotoxicity and Biocompatibility

**Figure 5:**
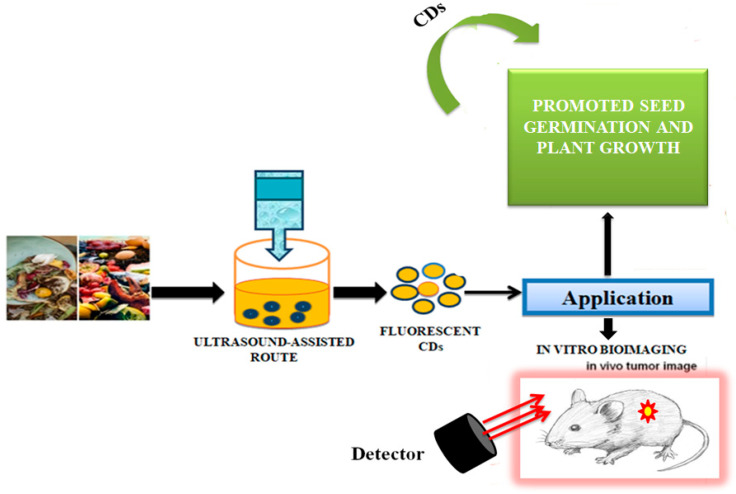
Synthesis of carbon dots from the waste food. The CDs were successfully used for in vivo tumour imaging, as described in [104].

CDs synthesised from green routes, using cost-effective and renewable sources, such as fruits, vegetables, and different types of waste are excellent alternatives. The unique properties of those CDs, such as good biocompatibility, low toxicity, high-water solubility, catalytic behaviour, and electrical conductivity make them useful as biosensors in biomedical applications, especially in biolabelling, DNA-sensing microbial control, and drug-delivery studies, metal sensing, drug delivery, and energy storage [100,101]. Jhansi et al. [102] studied the biocompatible properties of CDs through an in vitro cytotoxicity test on L6 normal rat myoblast cells by using a 3-(4,5-dimethylthiazol-2-yl)-2,5-diphenyl-2H-tetrazolium bromide (MTT) assay. Arul et al. [103] reported that fluorescent nitrogen-doped CDs (N-CDs) could be prepared from kiwi fruit (*Actinidia deliciosa*) extract by a one-step hydrothermal method using aqueous ammonia. Due to low cytotoxicity, N-CDs were tested against L-929 (Lympho blastoid-929) and MCF-7 (Michigan Cancer Foundation-7) cells and interestingly, they exhibited anticancer activity. N-CDs also catalysed the degradation of carcinogenic agents such as rhodamine-B (RhB), a xanthene-based dye released from paper, textiles, paint, and leather industries, being applied as an illegal food additive, staining fluorescent dye and as a tracer dye using NaBH_4_. Park et al. [104] proposed simple adjustable experimental conditions such as ultrasonic power and the optimal proportion of solvents and reaction time for a large-scale preparation of water-soluble CDs from food-waste-derived carbon sources. The ultrasound-assisted route led to the production of 120 g CDs produced from 100 kg of food waste mixture, which were highly water soluble, photostable, photoluminescent, and with low cytotoxicity, and which could be used for in vitro bioimaging and promotion of seed germination and plant growth (Figure 5).

**Figure 6:**
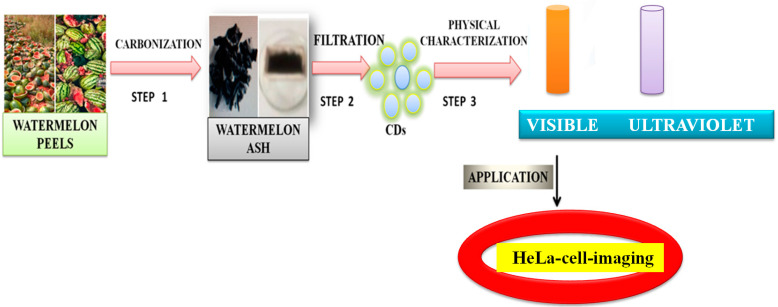
Synthesis of carbon dots from the watermelon peels. The CDs were successfully applied to HeLa cell imaging, as described in [105].

**Figure 7:**
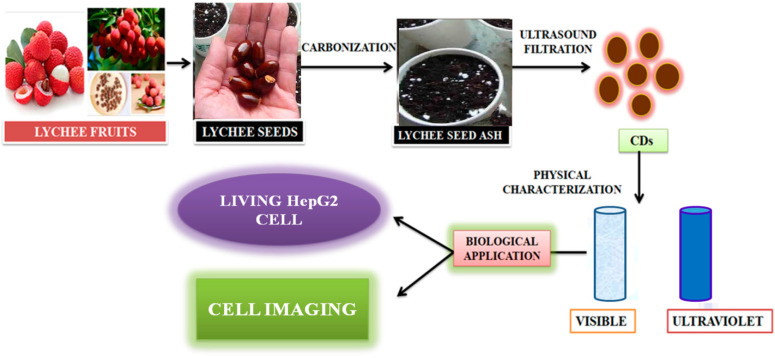
Synthesis of carbon dots from lychee seeds. The CDS were used for fluorescence imaging of living HepG2 cells, as described in [106].

Zhou et al. [105] achieved large-scale production of CDs by pyrolysis of watermelon-peel waste under a low temperature and followed by filtration. The obtained CDs showed strong-blue luminescence, excellent water solubility, good stability in solutions with a wide range of pH and high salinity. The as-prepared carbon dots were successfully used in HeLa cell imaging (Figure 6). Similarly, CDs with low inherent cytotoxicity could also be produced from lychee seeds by pyrolysis, and CDs with a Φ of 10.6% could be used in fluorescence imaging of living HepG2 cells (Figure 7) [106].

**Figure 8:**
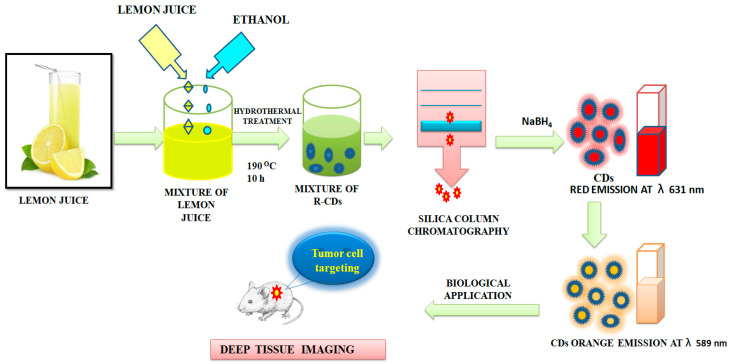
Synthesis of carbon dots from the lemon juice. The CDs were used for deep-tissue imaging, as described in [107].

Ding et al. [107] reported the preparation of red-emitting C-dots (R-CDs) from lemon juice using a hydrothermal method, followed by purification using silica column chromatography. The CDs exhibited Φ of 28% and an excitation-independent red emission maximum at 631 nm could be observed. The reduced R-CDs showed very negligible cytotoxicity. The surface states of R-CDs were crucial. The reduced R-CDs showed orange emission maxima at 589 nm and could be used in deep-tissue imaging as represented in Figure 8. Oh et al. [108] and Bilal et al. [109] reported the metacellular analysis of QDs. Table 1 lists cytotoxicity and the detection-limit range of a number of QDs and CDs to depict an overall scenario. Although due to the variation in the preparative method and precursors, it is difficult to have a realistic comparison, this would serve as a screenshot of the development in this arena.

## 5. Surface Functionalization

Multiple functional groups residing on the surface of CDs are accountable for their inherent properties. There are several ways to enhance the sensing ability and characteristic properties of CDs by functionalization: elemental doping (introduction of impurities, or heteroatoms) into CDs, forming a composite with appropriate matrix and surface passivation [121]. The enhancement of sensing through doping and the formation of composites (combination of more than one type of materials) are discussed here.

### 5.1. Doping of “N” Atom into CDs

**Figure 9:**
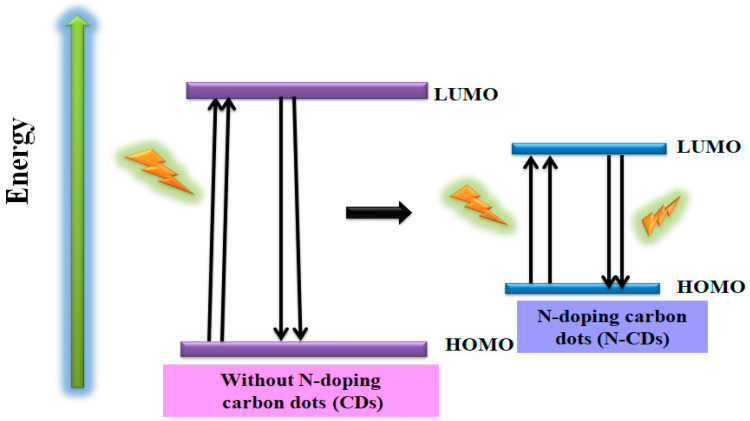
Energy diagram of CDs without doping and with N-doping.

Doping of heteroatoms (especially N, but also S, among others) in CDs is a frequently used route to enhance their physiochemical properties. Without N-doping, CDs have a large HOMO–LUMO gap. With N-doping, the same size of carbon and nitrogen ensures good interaction. The few valence electrons of N promote chelation with -NH_2_ and -COOH groups, present on the surface of CDs, which readily bind to the functional ligands (DNA, proteins, polymers, and organic molecules) via electrostatic, amidation and coordination interactions, inducing an upward shift in the Fermi level and electrons in the conduction band, and reduce the HOMO–LUMO gap to enhance the sensing properties of CDs. Therefore, N-doping in CDs lowers the gap between HOMO and LUMO and less energy is required for excitation and the sensing ability is increased [122,123] (Figure 9). The doping with nitrogen (aqueous ammonia) was confirmed by energy dispersive spectroscopy (EDS), as well as FT-IR spectroscopy, and the size was confirmed by HR-TEM [82].

#### 5.1.1. N-Doped CDs in “Chemical Sensing”

The development of selective, ideal, new, portable, cost-effective, highly sensitive chemical sensor and monitoring devices has directed attention to N-doped CDs. Liu et al. [124] introduced the chemical sensing ability of N-CDs towards nitrite (NO_2_^−^) under acidic conditions (pH 2.5) in a food sample. N-CDs were prepared by a facile hydrothermal carbonization method using *p*-phenylenediamine and citric acid as the nitrogen precursors. The surface of the resultant N-CDs consisted of multiple functional groups such as porphyrin CeNeC, amino N in N-(C) or HeN-(C) or sp^2^-hybridised C. Doped N was responsible for demonstrating the green photoluminescence (PL) emission maximum. Similarly, Liu et al. [125] prepared N-CDs using a green hydrothermal approach, followed by carbonization of alginic acid and ethylenediamine to measure Fe^3+^ in urban river water, within acidic mimicking conditions (pH 4). Chen et al. [126] introduced double atom doping, such as S, N-CDs, and successfully prepared doped CDs from a garlic green source, using the one-top hydrothermal method. The resultant S, N-CDs demonstrated excellent sensing ability towards Fe^3+^ present in lake and tap water, within the tough interferential environmental circumstances. Yuan et al. [127] reported the superior PL properties of N-CDs obtained from maleic acid and ethylenediamine through a hydrothermal method. They exploited their excellent sensor tendency to recognize the *p*-nitrophenol (4-NP) environmental water samples (4-NP is used as raw material in chemical industries, such as pesticides, pharmaceuticals, dyes, explosives, and leather). Shamsipur et al. [128] studied the ultrasensitive colorimetric sensing properties of N,P-CDs prepared by a green hydrothermal route and reported excitation-dependent PL emission behaviour. N,P-CDs were applied for the sensing of uranyl ions (UO_2_^2+^) in wastewater and hair samples. Lu et al. [129] showed that N-CDs synthesised using ultrasound techniques could detect Fe^2+^ ions in aqueous samples and inside a malignant tumour cell. N-CDs showed excellent PL quenching and temperature-dependent photoluminescence at 283–358 K for Fe^2+^ ions. Xie et al. [130] reported the synthesis of N-CDs following a green route from barley and ethanediamine using a hydrothermal method. Numerous hydrophilic groups were identified on the surface of N-CDs, which were responsible for the strong fluorescence properties at 480 nm. The N-CDs showed highly sensitive and selective sensing behaviour towards the most noxious and threatening heavy metal ion, Hg^2+^, at an ultratrace level of 10–160 µM. Latha et al. [131] proposed on–off fluorescent nanoprobes in the form of nitrogen-doped oxidized carbon dots (NOCDs), hydrothermally synthesised for the selective analysis of methanol (MeOH) adulteration in alcoholic beverages. NOCDs have good sensitivity toward MeOH and ethanol (EtOH). More than 90% of the fluorescent emission intensity of NOCDs decreased when 1% MeOH was present in distilled water, while intensity lowered by only 20% in the case of EtOH. Zhai et al. [132] reported the hydrothermal preparation of N-CDs, using garlic skins as a natural source, with 9% Φ. The resultant N-CDs showed huge potential as small fluorescent inks and films, and also for Fe(III) detection. Gu et al. [133] described the hydrothermal preparation of N-CDs using wolfberry with 22% Φ. Obtained N-CDs were successfully applied for the detection of Fe(III) and L-ascorbic acid (AA) with a limit of detection (LOD) of 3 µmolL·L^−1^ and 1.8 µmolL·L^−1^, respectively. John et al. [134] reported hydrothermal fabrication of N-CDs from Ruta graveolens with 18% Φ. The resultant N-CDs served as an effective fluorescent sensor for tetracycline (TC) with a LOD of 0.28 nM. Rong et al. [135] reported the preparation of N-CDs using the solid-phase pyrolysis method using guanidium chloride and citric acid as the catalyst. The N-CDs were used for the determination of Fe^+3^ with a LOD of 100 nM. Chai et al. [136] proposed the fabrication of N-CDs from the traditional Chinese medicine “Gastrodia elata”, with chrysanthemum as a catalyst, using a hydrothermal method, which was applied for the detection of phydroxybenzaldehyde (PHBA) with a LOD of 63 nM. Jia et al. [137] described the preparation of fluorescent N-CDs, following a one-step pyrolysis method, from black soya beans with 38.7% Φ. The obtained N-CDs showed dual responsive behaviour towards free radical scavenging and Fe^+3^. Li et al. [138] proposed the synthesis method of yellow–green emitting N-CDs using 1,2 diaminobenzene as a carbon and diaminobenzene as an N doping source, with a hydrothermal method. The N-CDs were used as fluorescent probes for the sensitive determination of Ag^+^ with LOD of 5 × 10^−8^ mol/L. Dang et al. [139] reported the preparation of fluorescence turn off on nanosensors in the form of N-CDs using a hydrothermal method, using citric acid and ethylenediamine as a carbon and nitrogen source, respectively. The N-CDs were used as a label-free sensing probe for the ultrasensitive detection of Cu^+2^ and ciprofloxacin (CIP), with LOD of 0.076 nM and 0.4 nM, respectively. Rao et al. [140] developed N-CDs via a microreactor using porous copper fibres with 73% Φ. The resultant N-CDs showed great potential for the detection of environmental hazard material, Hg^+2^ with LOD of 2.54 nM. Fu et al. [141] reported the preparation of N-CDs via the low-temperature approach using glassy carbon electrode (GCE) via dipping. The N-CDs were used for the detection of hydrogen peroxide (H_2_O_2_) and paracetamol with LOD of 157 nM and 41 nM, respectively.

#### 5.1.2. N-Doped Carbon Dots in “Biosensing”

Xu et al. [142] proposed a new carbon precursor of chrysanthemum for the synthesis of N-CDs, using hydrothermal pyrolysis. The surface of the resulting N-CDs showed distinct -COOH groups and pyridyl nitrogen atoms responsible for acidophilic fluorescent sensor properties. These novel fluorescent probes were applied for the label-free sensitive and selective measurement of H^+^, Fe^3+^ in a strong acidic medium and also for hydrazine detection. Sun et al. [143] introduced the yellow-emitting CDs (Y-CDs) with excellent fluorescent stability at different temperatures and pH conditions. These Y-CDs with good Φ of 16.7% were fabricated by a solvothermal approach and used for the successful detection of adequate cyanocobalamin, also known as vitamin B12, and folate protection, as well as cell imaging (MDA-MB-231 cells). Yu and coworkers [144] reported Saccharomyces fabricated N-CDs with Φ of 16%, obtained using a simple reliable one-pot microwave-assisted hydrothermal method. Those CDs served as excellent PL multifunctional nanobiosensors for pH detection and vitamin B12 probing, and their efficiency increased with a pH reduction from fourteen to two. Simoes et al. [145] studied the vital role of S/N-doped CDs as sensor and sensing probes, with negligible toxicity, for the detection and monitoring of different types of pesticides (insecticides, herbicides, fungicides, etc.) used in the protection of crops from harmful organisms, such as insects, rodents, fungi, weeds, and other pests. Tammina et al. [146] proposed an efficient means to analyse dopamine (DA) and temperature in an aqueous environment, employing N, P doped CQDs as sensitive and reversible sensors, prepared from citric acid, ethylenediamine and urea phosphate as carbon, and nitrogen precursors, respectively, by using a microwave digestion method. DA is the most important neurotransmitter in the human nervous system. Before the circulation in the body, DA is first collected in the brain to maintain learning, awareness, and blood pressure. Hence, DA detection plays a crucial role in the diagnosis of Parkinson’s disease. N,P doped CDs showed excellent sensing behaviour towards DA in the temperature range of 10–70 °C. Yola et al. [147] presented graphitic carbon nitride (g-C_3_N_4_) N-doped CDs composite (g-C_3_N_4_/CD_S_) with low toxicity and good sensitivity. The g-C_3_N_4_/CD_S_ were used for the recognition of neurotransmitter mediators in the central nervous system, such as epinephrine and drugs in pharmaceutical and clinical fields, at low concentration levels in urine samples. They could successfully overcome the shortcomings of traditional sensors (such as high cost, complicated pretreatment of sample, and time-consuming analysis) of capillary electrophoresis, chemiluminescence, fluorimetry, and high-performance liquid chromatography.

### 5.2. Carbon Dot Composites

**Figure 10:**
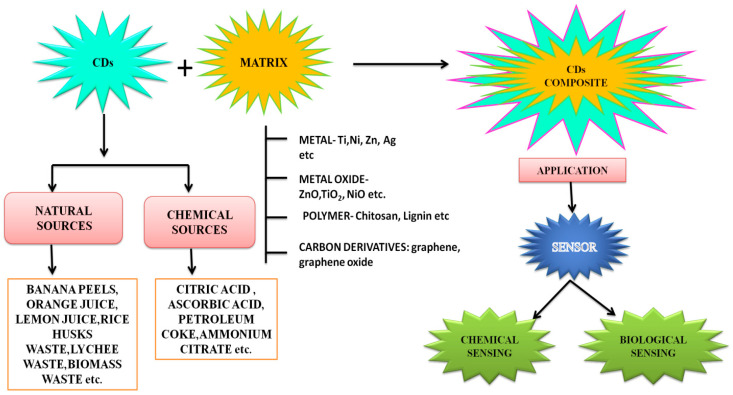
Overview of the synthesis of CD composites from various sources.

There are many examples of sensors using nanostructures. Many functional nanomaterials or matrices have been successfully integrated with CDs to develop composite probes. These matrices are metal NPs, metal oxides, bismuth-based metal compounds, polymers, and carbon materials, resulting in the formation of metal CDs, metal-oxide CDs, polymer CDs, and carbon-based CDs composites, as summarised in Figure 10.

#### 5.2.1. Synthesis of CD Composites

**Figure 11:**
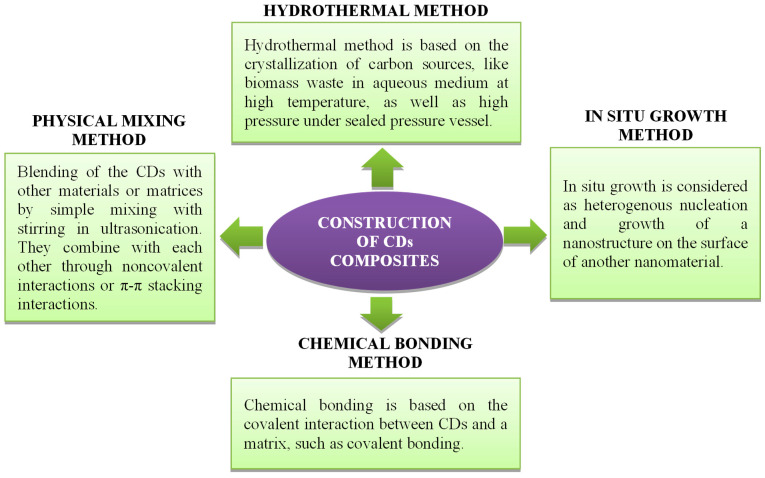
Synthesis of metal-based CDs composite.

CD composites can be prepared by different methods such as hydrothermal, physical mixing, in situ growth, and chemical bonding, as shown in Figure 11.

#### 5.2.2. Metal-Based CD Composites

Metal-based CD composites involve both metal and metal-oxide CDs and can be applied for chemical sensing as well as biological sensing of analytes. Fluorescent CDs hybridised with metal NPs were achieved mainly through nucleation and (in situ) growth of metal NPs. Because of oxygen-related functional groups, such as -OH, reducing agents are stabilised with the reductive group present on the surface of the CDs, and the reduction of noble metal ions assists the formation of CDs and metal NPs composites. Composites of this kind show incredible technological importance in sensing, fuel cells, optoelectronics, light-emitting diodes, fluorescent inks, photocatalysts, and heterogeneous catalysis [148,149]. The CDs formed composites with Au NPs and Ag NPs, which showed excellent photocatalytic abilities due to their surface plasmon resonance (SPR) absorption [150]. In recent years, metal oxide composites have attracted considerable attention in various fields, such as photonics, agriculture, electronics, medicine, and cosmetics [151,152]. They have also been successfully used in selective oxidation, acid-base catalysis, polymerisation, redox processes, hydrogenation and in many environmental applications for the elimination of contaminated elements.

**Figure 12:**
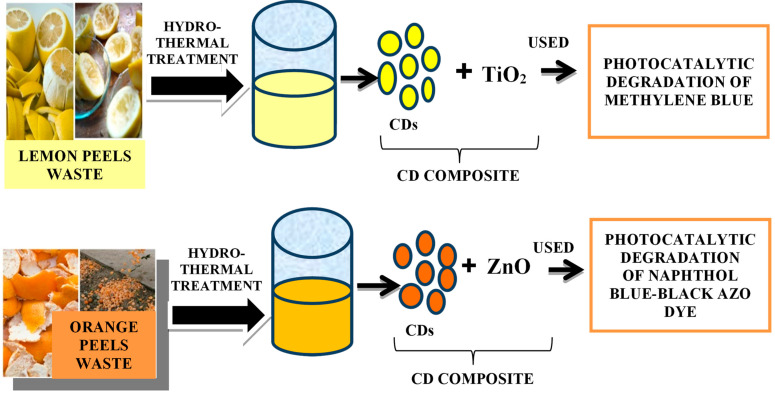
Synthesis of CDs composite from (**top**) lemon peel waste [158] and (**bottom**) orange peel waste [157] and their application for photocatalytic degradation of dyes.

As discussed earlier, CDs also have excellent biocompatibility, low cytotoxicity, strong chemical inertness, easy functionalization, and high resistance to photobleaching. However, after the formation of the composites with metal oxides, the photocatalytic behaviour was significantly enhanced [153,154,155,156]. Prasannan et al. [157] synthesised CDs from orange peel waste by a one-pot hydrothermal carbonization under mild conditions, and prepared composites with ZnO to enhance the efficiency of photocatalytic degradation of naphthol blue-black azo dye, under UV irradiation, where 84.3% degradation occured within 45 min (Figure 12, top). Tyagi et al. [158] synthesised CDs from lemon peel waste by a facile hydrothermal process. The resulting material showed spherical morphology and oxygen-rich surface functionalities, being used for the detection of Cr^6+^ with a LOD of 73 nM. CDs formed composites with TiO_2_ for the photocatalytic degradation of methylene blue under UV light irradiation (Figure 12, bottom).

CD metal-oxide composites show characteristics of a tremendous electron transfer and reservoir due to their conjugated π structure. Some of the remarkable metal oxide NPs used are: TiO_2_, CuO, ZnO, PdO, and Fe_3_O_4_. Syntheses involve chemical, physical, and biological methods and recently chemical pathways including mechanochemical processes, surfactant precipitation, sol-gel, solvothermal, hydrothermal, and emulsion methods have been popular [159,160,161].

#### 5.2.3. Metal-Based CD Composites Used in “Chemical Sensing”

Sohal et al. [162] designed and prepared CD-MnO_2_ nanosphere composites by mixing CDs with MnO_2_ nanospheres of different sizes. The CDs were synthesised by a microwave method using ascorbic acid as a precursor, while MnO_2_ nanospheres of different sizes were prepared by adjusting the concentration ratio of methionine and KMnO_4_ at room temperature. CD-MnO_2_ composites served as sensors and displayed excellent fluorescence intensity, high Φ, economic viability, and ecofriendliness and could successfully overcome the limitations of conventional methods for the detection of glutathione (GHS). The composites were found to be efficient for the rapid detection of GSH even at very small concentrations and were applied in various areas, such as the food, health, pharmaceutical, and cosmetics industries. CD decorated MgO nanocomposites were prepared by a hydrothermal method and used as high sensitivity new Schottky sensor devices for the detection of various reducing gases, such as H_2_S, even at low concentrations [163]. H_2_S is widely used in chemical laboratories and industrial sectors; however, it is very difficult to detect H_2_S, due to its colourless, tasteless, and odourless nature. This may cause the uncontrolled release of H_2_S, which results in an immediate collapse of the nervous system, with difficulties in breathing, and often a high death rate.

Transition metal oxides, in general, show good photocatalytic behaviour and have several photocatalytic applications. However, recent research has shifted the focus to binary metal oxides, such as ZnFe_2_O_4_, CuBi_2_O_4_, and CuMn_2_O_4_, due to their low cost, magnetic separability, superior electrical (narrow bandgap) and optical (photocatalytic activity) properties. Interestingly, the properties of the binary metal oxides were remarkably enhanced once they were combined with CDs.

Woo et al. [164] used an ionic liquid (IL) to synthesise CD-embedded cellulose transparent films, using a one-step solution synthesis process. The material prepared showed excellent transparency, great photostability, and good reusability properties. It could be used for the detection of Fe^3+^. The synthesis of multifunctional magnetic ferrite (MFe_2_O_4_, where M = Mn, Co)-molybdenum disulfide (MoS_2_) CDs nanohybrid composites was reported by Wang et al. [165] using a one-step solvothermal method, in which fluorescent CDs were embedded in MoS_2_. The abundant hydroxyl groups on the surface of MoS_2_ nanosheets with large surface areas and superior mechanical flexibility were retained in MoS_2_ nanosheets and CD nanocomposites, where metal ions (Fe^2+^, Mn^2+^, Co^2+^) were used as precursors. The introduction of magnetic ferrite MFe_2_O_4_ not only gave good recycling potential but also further enhanced the removal efficiency of metal pollutants, such as Pb(II). These composites could be successfully used in gas sensing, especially in the estimation of environmental pollutants. Yu et al. [166] used CDs-hexadecyltrimethyl ammonium bromide (CTAB)-chitosan (CS) composites as modified electrochemical sensors for the determination of 2,4-dichlorophenol (2,4-DCP), which is a potentially carcinogenic and toxic chlorinated phenol employed in pharmaceuticals, pesticides, and fungicides, causing serious water pollution. These simple and ecofriendly composites formed by combining CDs with CTAB and an adsorbent, as electrode modifiers, increased the phenol contents on the electrode surface and CS through electrostatic self-assembly. A cost-effective sensor with fast response and ease of operation was obtained.

The preparation of QDs/CDs@ zeolitic imidazolate framework-8 (ZIF-8) composites was reported by Ma et al. [167]. They used the composites such as ratiometric fluorescent (RF) sensors for measuring the concentration of Cu^2+^ ions in tap water. Several industries release unusually high amounts of Cu^2+^ ions, enough to be able to cause severe diseases, such as Wilson’s disease, neutropenia, Alzheimer’s disease, etc. QDs/CDs@ZIF-8 composites with excellent sensing properties can potentially overcome the shortcomings of spectrometric and surface characterisation techniques.

Cheng et al. [168] investigated the gas sensing behaviour of hierarchical litchi-like In_2_O_3_/CDs composites and reported them as excellent sensors toward harmful, toxic nitrogen dioxide gas (NO_2_) released from liquid laundry detergents, vestige fuels, vehicle exhaust, etc. The composites were hydrothermally prepared, and their gas sensing performance was evaluated by analysing the heterojunction interaction between CDs and metal oxide of In_2_O_3_/CDs and pristine In_2_O_3_.

Metal-organic framework (MOF)-based fluorescence chemical sensing devices were used by Yang et al. [115] for the detection of 4-NP produced from dyes, leather products, and pharmaceuticals industries. The MOF-based composites were formed by the integration of amine-passivated CDs into zirconium-based MOF-66, by a postsynthetic modification approach. Similarly, CDs@Eu-MOF was used as dual-emission ratiometric fluorescent probes, with a detection range of up to 300 μM [169]. The composites were prepared by a green hydrothermal method and could be used for the estimation of Hg^2+^ as well as Eu^3+^ in water samples. Lin et al. [170] studied branched poly-(ethylenimine)-capped CDs-ZIF-8 composite as a fluorescent MOF sensor for the analysis of Cu^2+^ ions in water samples, by exploiting their high-adsorption capacity. Liu et al. [171] prepared dual-emissive sheet-like CDs-embedded Ag@EuWO_4_(OH) luminescent nanocomposites fabricated from the Eu(NO_3_)_3_.6H_2_O-(NH_4_)10H_2_(W_2_O_7_)_6_•*x*H_2_OCS(NH_2_)_2_ where thiourea was used as a carbon source. The hydrothermal method, in association with photochemical deposition, yielded the composites. Upon irradiation with UV light, Ag^+^ was reduced to Ag NPs, which were deposited on the surface of EuWO_4_(OH), by an in situ photochemical deposition method to form CDs embedded Ag@EuWO_4_(OH) composites. These could be used for effective ratiometric determination of hydrogen peroxide (H_2_O_2_), based on the opposite response of emission intensities of Eu^3+^ (with emission lines at 614 nm) to ensure the safe use of H_2_O_2_. The concept of “kill waste by waste” was applied by Mehta et al. [172] through the synthesis and design of CQDs/TiO_2_ nanocomposites. The composites could be used for the assessment of toxic heavy metal ions and photodetoxification of industrial dyes in wastewater, under visible light. CDs@SiO_2_@ CdTe QD hybrid spheres were reported by Rao et al. [173] to be used as ratiometric sensors for the analysis of Cu^2+^ in vegetable and fruit samples.

#### 5.2.4. Metal-Based CD Composites in “Biosensing”

There are a number of success cases using metal-based CD composites in biosensing and a large portion of these cases are promising. For example, Qin et al. [174] reported that Eu^3+^/CDs@MIL-53 have a highly selective detection ability towards toluene diisocyanate (TDI), an aliphatic strong irritant that leads to asthma, as well as diaminotoluene (TDA), the measurable metabolite of TDI in urine. The biomarker was obtained by mixing CDs and lanthanide ion Eu^3+^ in a MOF, to generate a newly ratiometric fluorescence hybrid probe. The preparation of a photoelectrochemical (PEC) biosensor was reported by He et al. [175]. Authors used in situ integration of CDs into secondary anodized TiO_2_ nanotube arrays (TiO_2_ NTAs), with the help of a hydrothermal approach, followed by the immobilisation of glucose oxidase on the surface of the composite. The PEC biosensor exhibited conductivity, good biocompatibility, strong light absorption, and reflection ability in visible light for sensitive and selective detection of glucose.

A DNA detection sensor in the form of a Pd-Au@CDs nanocomposite modified glassy carbon electrode (Pd-Au@CDs/GCE) was developed by Huang et al. [176]. The CDs prepared from green banana peels sourced using microwave treatment were mixed with Pd-Au NPs through a sequential-reduction method. The resultant composite was highly sensitive, compared to other reported electrochemical biosensors for measuring colitoxin DNA in human serum, after treatment with a carboxyl ammonia condensation reaction to immobilize a single-stranded probe DNA. Yang et al. [177] reported the synthesis of a thiol functional group containing ferrocene derivative (Fc-SH) mixed with Au NPs/CDs (Au/CNC) nanocomposites. The material was stabilised after coupling with graphene-modified GCE to obtain Fc-S-Au/CNC/graphene/GCE by an electrochemical method. The composites were used in the ultrasensitive and direct detection of dopamine, ascorbic acid, acetaminophen, and uric acid. They were useful for the prevention and treatment of many diseases, such as colds, mental illnesses, infertility, cancers, acquired immunodeficiency syndrome (AIDS), etc.

The design and preparation of CDs-MnO_2_ nanosphere composites were reported by Sohal et al. [162], by combining CDs with MnO_2_ nanospheres of different sizes. The composites were used as sensors with excellent fluorescence intensity and high Φ. Perelshtein et al. [178] reported, for the first time, the synthesis of iron-carbon dot nanohybrid-Fe@CDs composites, using an ultrasound-assisted approach, in which air-stable, highly magnetic (metallic) iron nanoparticles were used as precursors, and significantly fluorescent CDs synthesised from polyethylene glycol (PEG-400) were used as the carbon source. The composite had excellent fluorescent and magnetic properties and could be used as a sensor, probe, and detection tool in bioimaging applications based on magnets, such as specific antibodies, cholesterol, uric acid detection in human blood serum and also in the detection of diseases, such as cancer and tuberculosis, among others.

A ZnO@N-C hybrid was also decorated by mixing N-CDs prepared using a direct facile hydrothermal method with ZnO nanoparticles [179]. The composite exhibited excellent biocompatibility, low cytotoxicity, and good PL and photocatalytic activity and, hence, could be used as a photocatalyst for the degradation of cytotoxic pollutants, such as methylene blue. The N-doping on the CDs also allowed their application as multicolour fluorescence probes in biomedical applications. Huang et al. [180] reported the use of cuprous oxide-carbon dots/Nafion (Cu_2_O-CDs/NF) composite as a biosensor for the selective and consistent determination of DNA content in human serum. Zhai et al. [181] introduced a latent fingerprint and fluorescence labelling marker in the form of CDs@montmorillonite (g-CDs@MMT) composites under 405 nm radiation. The composites were fabricated by intergrading green emissive CDs into a MMT clay matrix and could be applied for the recognition of the surfaces of various types of objects.

Silver-CD (Ag-CD) nanocomposites were also prepared by mixing fluorescent CDs with a reduced silver ion (Ag) leading to a unique peroxidase-like composite. The material induced the oxidation of 3,3′,5,5′-tetramethylbenzidine (TMB) into oxidized TMB (oxTMB), in the presence of H_2_O_2_, since CDs transfer their electrons to H_2_O_2_ and generate hydroxy radicals. The oxTMB was detected by the blue colour, with an absorption maximum of 652 nm. Hence, Ag-CDs nanocomposites were used to accurately detect uric acid (UA) by applying a reducing agent to reduce the blue oxTMB into colourless TMB [182].

Wang et al. [183] designed a unique fluorescence resonance energy transfer (FRET)-based biosensor by using fluorescent CDs with MnO_2_ nanosheets, in the form of a superior energy donor–acceptor pair, which showed high selectivity and sensitivity for the recognition of glutathione in whole human blood samples. A MOF-based biosensor was fabricated by Xu et al. [184] for the selective detection of plant pigment quercetin (QCT) in the form of CD-embedded MOF@molecularly imprinted polymer nanoparticles (CDs@MOF@MIP), where CDs served as signal transducers. The CD-doped Fe_3_O_4_ embedded g-C_3_N_4_ nanosheets (CDs/Fe_3_O_4_@g-C_3_N_4_) composite serves as a rapid, easy, and accurate electrochemical sensing platform to detect thiocyanate (SCN^−^) even at a trace level [185]. Prior to the synthesis of the composite for the detection of SCN^−^, suitable approaches are needed since even a very small amount of SCN^−^ causes severe adverse effects on the human body, such as cardiovascular threats, disturbance on protein dialysis, increasing the possibility of cancer, and also leading to hypo- or hypersecretion of iodine via the thyroid glands. The obtained composite performed well as a sensing device in medical diagnosis and ecological monitoring.

A gold nanoparticle-CD core-shell (Au@CDs) nanocomposite also served as both a colorimetric and fluorometric selective and sensitive sensor for consistent diagnosis of biomolecules, containing thiols, such as proteins, enzymes, amino acids, and peptides [186]. CDs/Zn(OH)_2_ composites prepared using a template-free microwave-assisted approach were successfully used as ultrasensitive sensors for the detection and removal of the heavy metal ions Cu^2+^ or Hg^2+^ in water samples, either in online or offline modes [187]. Wang et al. [188] reported the synthesis of NiAl layered double hydroxide (NiAl-LDHs) composites by mixing CDs into flower-like NiAl-LDHs. The resultant material was highly selective and thus could be used as a nonenzymatic electrochemical biosensor for the detection of neurotransmitters, such as acetylcholine.

A responsive electrochemiluminescence (ECL) immunosensor was also developed in the form of nanoporous silver (NPS), that is, NPS@CDs composite [189]. It was prepared by loading CDs (obtained from electrooxidation of graphite and used as ECL reagent) into NPS with a controllable 3D structure. This immunosensor could be used as a cancer marker and prostate protein antigen in clinical diagnosis.

The differences of the cytotoxicity and detection limit in CDs, N-CDs as well as CDs composite are summarised in Table 2.

## 6. Conclusions

Here the rapid advancement of CDs in the field of sensing has been critically reviewed. The need for the development of CDs, in substitution of QDs, was identified and efficient means to tune the properties of CDs, including heteroatom doping and the formation of CD composites, were highlighted. The CDs appeared to be promising carbon materials, compared to traditional QD materials, due to their inherent properties, such as low toxicity, biocompatibility, water solubility, cell-membrane permeability, good fluorescence emission, and excellent photocatalytic efficiency.

However, there were shortcomings which restricted their direct use as sensors. The need for improving the properties of CDs for sensing applications pointed to the functionalization with heteroatoms, as well as the preparation of nanocomposites in combination with matrices, such as metals, metal oxides, polymers, or carbon-based materials, or surface passivation and doping of heteroatoms.

The CDs were fabricated using both top-down and bottom-up approaches. Among them, the bottom-up approach gained more attention because of its simplicity, low or no cost, high yield, and ecofriendliness. Due to their negligible toxicity and good biocompatibility originating from multiple functional groups, CDs demonstrated amazing impacts on the optical properties and fluorescence phenomenon leading to familiar applications in the biomedical fields, such as bioimaging, biosensor, nanomedicine, and drug delivery (as a biosensor) as well as detecting a variety of chemical species with applicability in practical sample analysis found in environmental sources such as water bodies (as a chemical sensor).

We first described N-doped CDs and explored their use in both chemical sensing and biosensing. Furthermore, the potential of metal-based CDs composites in chemical sensing as well as biosensing of environmental resources and in the human body was discussed. N-CD and metal-based CD composites served as biosensors and chemical sensors for the detection and monitoring of certain type of diseases, such as infections, cardiovascular diseases, neurodegenerative disorders, etc.

For successful recognition and quantitative estimation even at trace levels, selectivity and sensitivity still remain an issue and concerted efforts still need to be made to develop chemical and biosensors based on N-CDs and metal-based CD composites, aiming towards the enhancement of affinity and quenching frequency, while retaining the inherent properties of CDs. This review may serve as an informative platform to draw the attention of chemists, biologists, physicist, industrialists, medical professionals, and material scientists to work together for the development of efficient CD-based chemical and biosensors for real life applications.

## Figures and Tables

**Table 1:** Cytotoxicity and detection-limit range of QDs and CDs.

Quantum Dots vs. Carbon Dots	Source	Method	Cytotoxicity and Detection-Limit Range	Reference
CdTe QDs	CdCl_2_, NaBH_4_, tellurium powder, phosphate buffered saline tablets	Electrochemical method	118 ± 49 μg/mL with the help of electrochemical assay 157 ± 31 μg/mL by MTT cytotoxicity assay	[110]
InPZnS QDs	InPZnS alloy core and a thin ZnS shell	Heating Up Method	70 nM of QDs responsible for cytotoxic effect on environmental health on 72 h exposure	[111]
Indium-based QDs	In and Zn content and implementing a more robust outer shell	Heating Up Method	12.5 mg/kg and 50 mg/kg QDs generally collected in the liver as well as the spleen	[112]
InZnP and InZnPS QD	Indium myristate, ZnSt_2_	Precipitation Method	6.25–200 nM upon 24 h exposure	[113]
CDs	Onion waste	Hydrothermal method	0.31 μM	[114]
CDs	ZrCl_2_, trinitrophenol (TNP), 2,4-dinitrophenol, 4-nitrophenol, phenol, Polyvinylpyrrolidone, acetic acid, citric acid	Hydrothermal Method	0.01–20.0 μM at a low-detection limit of 3.5 nM	[115]
CDs	Gelatin	Hydrothermal Method	1−75 μmol/L	[116]
CDs	Carbohydrate	Hydrothermal Method	0 to 1 × 10^3^	[117]
CDs	DEA	Microwave Method	5.0 × 10^−2^ to 8.0	[118]
CDs	Catechol	Hydrothermal Method	1 × 10^−2^ to 25	[119]
CDs	Citric acid	Hydrothermal Method	8.0 × 10^−2^ to 50	[120]

**Table 2:** Synthesis routes, source, and cytotoxicity of CDs, N-CDs, and CDs composites.

CDs	Source	Preparation Method	Cytotoxicity/ Detection Limit/ Sensor Response	Quantum Yield	Detection Element	Reference
CDs	Citric acid and urea	Hydrothermal method	23 mM	44.7%	Toxic Be^2+^ ions	[190]
CDs	Free Gum Tragacanth and Chitosan	Hydrothermal method	50 µg/mL	-	Cellular Biosensing	[191]
CDs	Carica papaya waste pulp	Hydrothermal Method	0.708–2.4 ppb	23.7%	Cr ions detection	[192]
N-CDs	Citric acid and ethylenediamine	Microwave-assisted solvothermal method	0.089 µmol/L	79.6%	Hg^2+^	[193]
N-CDs	Citric acid and ethanediamine	Hydrothermal method	0.076 nM	84%	Cu^2+^ ions	[194]
N-CDs	Citric acid and ethanediamine	Hydrothermal method	84 nM	-	Fe^3+^ in aqueous solution	[139]
CDs@MgO nanocomposite	Magnesium nitrate dehydrate, KOH, HF, ethanol and D-glucose	Hydrothermal method	120 ppm	~74 ± 3%	H_2_S	[163]
Carbon dots loadedTiO_2_ nanocomposite	Diplocyclos palmatus leaf extract	Hydrothermal method	12.5 mM	54%	Fe ^3+^ and acute-hepatopancreatic necrosis disease (AHPND)	[195]

## Data Availability

Data will be made available upon request.
